# The Relationship Between the Level of Food Neophobia and Children’s Attitudes Toward Selected Food Products

**DOI:** 10.3390/nu17081347

**Published:** 2025-04-15

**Authors:** Łukasz Długoński, Magdalena Skotnicka, Marek Zborowski, Mateusz Skotnicki, Marcin Folwarski, Sabri Bromage

**Affiliations:** 1 Department of Commodity Science, Faculty of Health Sciences, Medical University of Gdansk, 7 Debinki Street, 80-210 Gdansk, Poland; dlugonski@gmail.com; 2 The Faculty of Medicine and Health Sciences, University of Applied Sciences in Nowy Sącz, Kościuszki 2G, 33-300 Nowy Sącz, Poland; mzborowski@ans-ns.edu.pl; 3 Students’ Scientific Circle of Pediatrics, Medical University of Gdansk, 80-210 Gdansk, Poland; mateuszskotnicki@gumed.edu.pl; 4 Department of Clinical Nutrition and Dietetics, Medical University of Gdansk, 80-210 Gdansk, Poland; marcin.folwarski@gumed.edu.pl; 5 Community Nutrition Unit, Institute of Nutrition, Mahidol University, 999 Phutthamonthon 4 Road, Salaya, Phutthamonthon, Nakhon Pathom 73170, Thailand; sabri.bro@mahidol.edu; 6 Department of Nutrition, Harvard T.H. Chan School of Public Health, 655 Huntington Avenue, Building 2, Boston, MA 02115, USA

**Keywords:** children, Children Neophobia Scale, attitudes towards

## Abstract

Background: Food neophobia, defined as the fear of eating new and unfamiliar foods, can influence the development of children’s eating habits and limit the variety in their diets. The present study aimed to assess the level of food neophobia in children based on parents’ opinions and to analyse their attitudes towards a variety of foods. Methods: The survey was conducted in the form of a questionnaire. The study was conducted in two stages. In the first stage, children’s level of neophobia was assessed using the Children’s Food Neophobia Scale (CFNS), which was completed by parents/guardians. A total of 133 participants representing paired responses were selected for analysis. In the second stage, children completed an original graphic questionnaire assessing their attitudes towards selected foods, divided into groups: positively, negatively, and neutrally perceived. The survey was conducted in a primary school in Pomeranian Province in January 2025. The Kruskal–Wallis test was used as a non-parametric statistical test to compare three groups. If necessary, post hoc tests (Dunn’s or Tukey’s) were performed to determine between which specific groups the differences existed. To assess the statistical relationship between the level of neophobia declared by parents and the type of attitudes among children, Pearson’s Chi2 test was used with a statistical significance level of *p* < 0.05. Results: It was found that 42.11% of children showed a high level of food neophobia, indicating a significant problem in the acceptance of new products in the diet. Commonly known and widely consumed products, such as ice cream, tomatoes, and cutlets, received the highest ratings. Edible insects and edible flowers were the least preferred. The Kruskal–Wallis test revealed significant differences in the level of neophobia among the three groups, and post hoc Tukey tests were conducted to determine the specific groups between which these differences occurred. Children with high and low levels of neophobia can exhibit both positive and negative attitudes toward different types of food. Conclusions: The results of the study highlight the importance of nutrition education for children and their parents to reduce fear of new products and promote more variety in the diet. Future research would benefit from examining the influence of family and peer environment on the development of food neophobia.

## 1. Introduction

Food neophobia is a phenomenon that can significantly affect children’s health and development. It poses a challenge to ensuring a diverse and nutritionally adequate diet, which is essential for proper growth and long-term well-being. Understanding the mechanisms of this condition is essential not only for prevention and nutritional education but also for shaping public health strategies that support healthy eating habits from an early age [1]. Food neophobia is an attitude characterised by an aversion to eating new foods and avoidance of trying unfamiliar foods. A key component of neophobic behaviour is a strong and persistent aversion and sometimes even fear of trying new foods. Individuals affected by this condition often lack the willingness to try new flavours, textures, or colours of food. Rejection may also extend to previously accepted foods that appear different due to changes in preparation or presentation [2]. Neophobic behaviour is most prevalent among children aged 2 to 5 years, a critical period for establishing lifelong eating habits that influence overall health outcomes. Due to limited knowledge of the phenomenon, many parents do not recognise neophobia in their children. Some studies suggest that the actual prevalence of food neophobia may be underestimated due to parental unawareness or misinterpretation of their child’s eating behaviour. Since neophobia often involves the rejection of nutrient-dense foods, underdiagnosis may negatively impact children’s dietary quality and long-term health outcomes [3,4]. According to Johnson et al., food neophobia is often perceived as a normal developmental phase. However, when prolonged or severe, it may lead to nutritional deficiencies and broader public health challenges. However, it can be a serious clinical problem, especially when exacerbated and associated with ongoing anxiety related to food consumption [5]. The first two years of life are crucial for the development of appropriate eating habits. Food patterns and preferences formed during this period often continue into preschool, school age, and adolescence [6]. Food neophobia may remain even during later adult life. Therefore, further research is needed to better understand the factors that may influence eating habits in adulthood [7]. A review by Lafraire et al. highlighted the distinction between picky eating and food neophobia. While picky eating involves selective refusal of certain familiar foods based on preference, food neophobia is characterised by a strong aversion to trying new or unfamiliar foods. Unlike picky eating, which can be influenced by taste or texture preferences, food neophobia stems from an inherent reluctance or fear of novelty in food choices. The underlying mechanisms behind these behaviours remain poorly understood. Four main causes for food rejection have been proposed: distaste, anticipated negative consequences, unattractive origin, and lack of recognition as edible [8]. Among the factors influencing children’s food choices, the most significant direct influence comes from preferences and habits shaped by their immediate environment. Children tend to prefer familiar, mild, and sweet foods, and aversion may occur towards unfamiliar foods [9]. It is worth noting that parents’ assessment of neophobia and selective eating in children is based on how parents perceive their child’s behaviour [10,11]. Neophobic behaviour is most common in children aged between 2 and 5 years, a critical period for establishing lifelong eating habits that influence overall health outcomes. Since parents play a central role in shaping their child’s food environment, their attitudes and feeding practices can either reinforce or mitigate food neophobia. Children tend to imitate their parents’ eating behaviours, dietary preferences, and overall approach to food consumption. Consequently, parental awareness and strategies are essential in addressing food neophobia effectively. The aim of this study was to evaluate the extent of food neophobia in children based on caregiver reports and to examine children’s attitudes toward different foods. Additionally, the study aimed to determine potential correlations between the degree of food neophobia and specific eating behaviours, providing insight into factors influencing dietary choices in early childhood.

## 2. Materials and Methods

### 2.1. Study Design

The study was conducted in two stages. The first stage of the study involved assessing the child’s level of food neophobia based on the parent’s or caregiver’s perspective. The second stage examined children’s attitudes toward new and unfamiliar food products. The parent survey utilised the Children’s Food Neophobia Scale (CFNS), while the child survey employed a proprietary graphic questionnaire designed to evaluate their attitudes towards various foods. The children’s questionnaires were administered in a public primary school in the Pomeranian Voivodeship, Poland, in January 2025. The parents’ questionnaires were filled in at home. The children’s graphic questionnaires and the CFNS questionnaires for parents were coded numerically, allowing for unambiguous identification and linking of responses between child and parent/guardian. Written consent for the child and parent to participate in the study was included. The survey was fully anonymous, and the results were used for research purposes only. Participation in the study was voluntary. Initially, the questionnaire and consent form were distributed to caregivers, and on the following day, children completed their questionnaires at school. Both stages of the survey were conducted in accordance with ethical principles, including non-maleficence, beneficence, justice, and autonomy, as outlined in the Declaration of Helsinki (2000). Personal data were anonymised in accordance with the European General Data Protection Regulation (RODO 679/2016). The study received approval from the Bioethics Committee of the University of Applied Sciences in Tarnów [8a/2025].

### 2.2. Level of Food Neophobia (FNS) of the Child According to the Parent

The first stage involved parents or caregivers completing a paper-based survey at home. The parent survey was based on the Child Food Neophobia Scale (CFNS). The survey included statements such as:

1. My child tries new and different foods all the time.
2. My child does not trust new foods.
3. If my child does not know what is in a food, I will not try it.
4. My child likes foods from different countries.
5. My child thinks ethnic foods are too strange to eat.
6. My child will try new foods.
7. My child is afraid to eat things he/she has never eaten before.
8. My child pays special attention to what he/she will eat.
9. My child will eat almost anything.
10. My child likes to try new ethnic restaurants.

The CFNS index includes ten items rated on a 7-point scale, where 1 means “Strongly disagree” and 7 means “Strongly agree”. A total of 10 to 70 points can be scored. Each question is assigned a value from 1 to 7, and the total score is calculated by summing all responses. Five items reflect neophilic attitudes (i.e., openness to new foods) and five items reflect neophobic attitudes. The consistency of the questions for the full sample for n = 133 was determined by Cronbach’s Alpha coefficient α = 0.77, which was considered satisfactory. The FNS score was calculated as the sum of the responses to the 10 questions. For the neophilic items, the scores were reverse-coded. A higher total score indicates a higher level of food neophobia. The children’s level of neophobia was categorised into three groups: neutral (score within mean ± 0.25SD), high (score > mean + 0.25SD), and low (score < mean − 0.25SD). Specifically, scores above 46 indicated high neophobia, scores between 39 and 45 indicated neutral neophobia, and scores below 38 indicated low neophobia. The questionnaire was developed based on the Food Neophobia Scale proposed by Pliner and Hobden [12].

Parents’ assessment of neophobia and selective eating in children is based on how parents perceive their child’s behaviour. Since parental perceptions can strongly influence children’s eating habits, it is essential to compare these assessments with the children’s actual attitudes towards different foods. This comparison provides insight into how caregiver-reported levels of food neophobia correlate with children’s preferences. Thus, the children’s attitudes were also assessed, aiming to explore how they relate to the parental assessments of food neophobia. The aim of this study was to assess children’s levels of food neophobia, reported by caregivers, and children’s attitudes towards different foods.

### 2.3. Assessment of Children’s Attitudes to Food

The next stage was a questionnaire, which was aimed at children and consisted of 12 pictorial questions focused on food preferences. Each item in the proprietary pictorial questionnaire was illustrated with standardised images of food items, designed to be age-appropriate and easily recognisable by children (Supplementary Material Figure S1). The visual format aimed to enhance understanding, reduce potential biases related to literacy skills, and increase engagement with the survey [13]. Food items were categorised into three groups: positively, negatively, and neutrally perceived, based on prior research and literature [14]. The survey was conducted in a controlled school setting, during a lesson, under the supervision of a teacher who provided technical assistance without influencing responses. The children were given sufficient time to complete the survey. The completion process was carried out individually, meaning that each child worked independently to avoid influencing each other’s answers.

The group of products perceived positively included: hamburgers, ice cream, ketchup, and pasta. The whole group of products was chosen because of their popularity and high availability, pleasant taste, and convenient way of preparation and consumption. These are products that are liked by children and are often associated with pleasure and convenience in everyday life [15].

Shrimps, rabbit, edible insects, and edible flowers were selected for a variety of cultural, moral, and health reasons for the group of products perceived negatively. Shrimps can raise concerns about disgust and environmental pollution. Similarly, insects are seen as an unconventional food source, arousing widespread revulsion. And rabbits, often treated as pets, cause moral controversy and emotional discomfort. Edible flowers, although visually appealing, can be perceived as exotic, unpalatable, and inedible, and their nutritional value is often questioned [16,17,18]. Products perceived neutrally include the tomato, cutlet, cheese, and egg, as they are widely accepted and widely consumed in many cultures without extreme emotion or controversy [19]. These are products that are usually frequently included in the daily menus of Polish children. In the study, children evaluated these items using iconic smiley face symbols: a smiling face (☻) representing a positive attitude, a neutral face indicating ambivalence, and a sad face representing a negative attitude. This approach aimed to facilitate comprehension and accurate responses. Similar symbolic scales have been validated and are widely used in paediatric populations to assess preferences, emotions, and attitudes [20]. However, this method has some limitations. It may oversimplify nuanced attitudes and may be influenced by children’s interpretation of symbols or the desire to please adults. Furthermore, while the scale supports intuitive responses, it does not provide insight into the underlying reasons for these attitudes. Despite these limitations, the approach remains a practical tool for use in large-scale research with young participants. Based on these results, children were classified and divided into three groups of attitudes towards food. After the response collection stage, the numbers of each selected “smiley face” for each product were summed up to estimate the overall trends in children’s attitudes. The next stage was an attempt to identify relationships between parents’ and children’s responses.

Children’s attitudes towards the different product groups were analysed based on the results obtained in the survey. Children’s attitudes were further evaluated using a point-based scoring system: 5 points for a smiley face, 3 points for a neutral face, and 1 point for a sad face. Each child assessed 12 products, which meant that the maximum possible score was 60 and the minimum was 12. A division into three groups was used to classify the participants. A positive attitude includes children whose scores exceed the value determined as the mean + 0.25 standard deviations (SDs). Negative attitude includes children whose scores are below the mean − 0.25 standard deviations (SDs). A neutral (ambivalent) attitude includes children whose scores are within the range of mean ± 0.25 standard deviations (SDs). The summed scores for each product allowed us to determine the level of acceptance among children [21].

### 2.4. Participants

The survey was conducted in January 2025 in the Pomeranian Voivodeship, in one of the municipal primary schools in Poland. A total of 177 children and 177 of their parents or guardians took part in the survey. The choice of this particular school was dictated by its diverse demographic and socio-economic profile, allowing for a sample that included children from different backgrounds. This approach was intended to increase the representativeness of the results and to allow analysis of the influence of different environmental factors on children’s eating attitudes. However, it should be noted that limiting the study to one institution may affect the generalisability of the results to the entire population of children in Poland. Therefore, future studies should consider including a larger number of schools from different regions and with different socio-economic profiles to better understand the complexity of food neophobia among children. Participation was voluntary, and all parent–child pairs who provided informed consent were included. No specific exclusion criteria were applied, allowing for a broad sample reflective of the school’s population. Initially, the questionnaire, along with the consent declaration from both the parent and the child to participate in the study, was distributed to the parents. On the following day, the children completed their pictorial questionnaire at school. Ultimately, 37 parents/guardians who declined to participate in the survey were excluded from the study. Additionally, responses from seven children had to be discarded due to incorrect or incomplete completion of the pictorial questionnaire. As a result, a total of 133 participants, forming parent–child response pairs, were included in the final analysis. The study group comprised children aged 6–10 years, including 78 girls and 55 boys. No exclusion criteria were applied, and all willing parent–child pairs were eligible to participate in the study.

### 2.5. Data Analysis

Analysis was performed using Statistica 12.0. The Kruskal–Wallis test was used as a non-parametric statistical test to compare groups. If significant differences were detected by the Kruskal–Wallis Test, post hoc tests (either Dunn’s or Tukey’s) were performed to identify which specific groups differed. Dunn’s test was used when comparing multiple groups without assuming equal variances, while Tukey’s test was applied for pairwise comparisons when equal variances could be assumed. To assess the statistical relationship between the level of neophobia declared by parents and the type of attitudes among children, Pearson’s Chi2 test was used with a statistical significance level of *p* < 0.05.

## 3. Results

### 3.1. Children’s Level of Neophobia as Determined Subjectively by Their Caregivers

The first stage involved a subjective assessment of the children’s level of neophobia completed by parents. In the analysed sample, the highest proportion of parents (42.11%) reported a high level of neophobia in their children.

The ambivalent group, representing a neutral level of neophobia, includes individuals who show mixed feelings or conflicting responses. Thirty-four parents indicated that their children are characterised by a neutral level of neophobia. Low levels of neophobia refer to the tendency to accept new products. Those with low levels of neophobia are open to change, willing to experiment, and try new things. Parents noted that their children showed distrust of new foods and stated that their child did not trust new foods (n = 56). In addition, parents often reported that if their child does not know what the food is made of, they do not want to try it, nor do they want to try foods they have not tested before. In children with low levels of neophobia, a characteristic approach to new foods was observed. Parents of these children often noted that their children were eager to try new and different foods. They emphasised that their child was constantly trying new foods (n = 43). In addition, these children showed an interest in foods from different countries and a willingness to try new foods. The Kruskal–Wallis test revealed significant differences between the three groups, and post hoc Tukey tests identified statistically significant pairwise differences. All pairwise comparisons between the groups showed statistically significant differences (*p* < 0.05) (Table 1).

Table 2 presents the results of the Child Food Neophobia Scale in three groups with different levels of food neophobia. The Kruskal–Wallis test indicates that for all 10 questions, there are statistically significant differences between groups I, II, and III (*p* < 0.05). To determine which specific groups differ for each question, a post hoc Tukey test was conducted.

For question FNS 10 (“My child likes to try new ethnic restaurants”), group I (low neophobia) differs significantly from both group II (neutral neophobia) and group III (high neophobia). Children with a low level of neophobia (group I) demonstrate a significantly greater willingness to visit new ethnic restaurants compared to the other groups. The difference between group I and group III is even more pronounced than between group I and group II.

A noteworthy finding emerges from the analysis of question FNS 1 (“My child tries new and different foods all the time”). There is a significant difference between group III (high neophobia) and group I (low neophobia), indicating that children with higher neophobia levels are less likely to try new foods. Additionally, group I differs significantly from group II in this aspect, suggesting a gradual increase in openness to new foods as the level of neophobia decreases.

The overall mean suggests a moderate level of food neophobia in the studied population, with distinct differences between the groups. Other questions highlight a clear pattern in which group I (low neophobia) consistently exhibits greater openness to new culinary experiences than both group II and group III.

### 3.2. Assessment of Children’s Attitudes Towards Food

**Figure 1:**
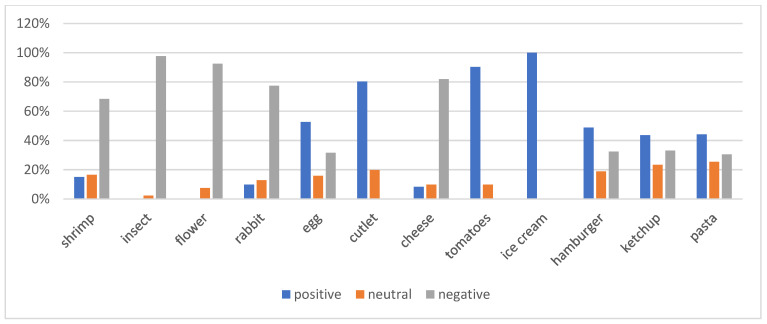
Children’s attitudes to food.

In the graphic questionnaire given to the children, it was necessary to express their attitudes towards 12 food products. The applied non-parametric Kruskal–Wallis test indicated statistically significant differences in the point ratings of 12 food products (*p* < 0.05). To determine which products differed significantly, Dunn’s post hoc test showed that in most cases, the differences in ratings were statistically significant. However, in a few product pairs, no statistically significant differences were observed: hamburger and tomato (*p* = 0.17), as well as hamburger and edible flower (*p* = 0.94). Additionally, no statistically significant relationship was found between ice cream and ketchup (*p* = 0.95) and cheese (*p* = 0.94). The results show that the largest group consisted of children with a positive attitude towards various foods, accounting for 44.36% of the total surveyed population (n = 59). This means that almost half of the children are open to trying different foods. However, it is important to note that these positive reactions varied considerably depending on the specific product; 100% of the children showed a positive attitude towards ice cream, a product commonly liked, while only 15.04% of the children reacted positively to prawns. Furthermore, none of the children reacted positively to edible insects (97.74% negative attitude) or edible flowers (92.48% negative attitude), indicating a clear aversion to less familiar and not very common foods (Figure 1). Children were grouped into three attitude categories based on total scores. The first characterised the negative attitude.

This group included responses with a total score of 12–33, where the lowest value was 14 points. A neutral attitude was shown by children whose responses ranged from 34 to 36 points, while a positive attitude was described when the total score exceeded 37 points. The study assessed children’s reactions to a variety of foods, ranging from the everyday and familiar, such as tomatoes and cutlets, to less typical and more exotic foods, such as flowers or insects (Table 3).

Children rated minced cutlet (4.47) and ice cream (4.20) most favourably. Our findings suggest that tomato (3.77) and hamburger (3.86) also ranked high in preference. The least liked products were edible insects (1.39), cheese (1.53), and prawns (1.78). The Kruskal–Wallis test, conducted based on attitude groups, revealed statistically significant differences in responses regarding hamburger, ice cream, ketchup, pasta, and tomato (*p* < 0.05) in Table 4. The test revealed statistically significant differences between the group characterised by negative attitudes and the groups exhibiting neutral and positive attitudes. In the comparison between the positive and neutral attitude groups, no statistically significant relationship was observed (*p* = 0.129), indicating that children with positive and neutral attitudes evaluated the level of food liking in a similar manner. The Tukey post hoc test determined which attitude groups exhibited significant differences for products identified as significant in the Kruskal–Wallis test. For the hamburger, a significant difference was observed between the negative attitude group and both the neutral and positive attitude groups. Similar results were found for the other four products (ice cream, ketchup, pasta, and tomato).

### 3.3. Relationship Between Levels of Neophobia and Children’s Attitudes

Pearson’s Chi-squared test revealed no statistically significant relationship between the level of neophobia and children’s attitudes towards the food products assessed by the children (*p* > 0.05); this indicates that CFNS scores do not predict actual food attitudes in children.

**Figure 2:**
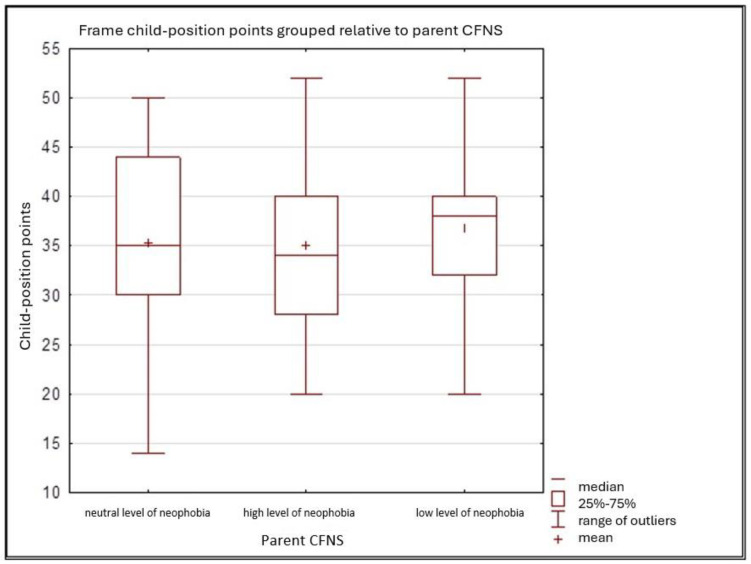
Children’s attitudes towards new foods in relation to parents’ levels of food neophobia as assessed by the CFNS.

The box plot (Figure 2) displays a box plot of children’s food attitudes according to CFNS-defined neophobia levels. The analysed groups of children are divided according to their level of neophobia into three categories: neutral level, high level, and low level. The graphical analysis shows no clear trend in the relationship between neophobia levels and attitudes toward different foods. The points are scattered, confirming the weak correlation between the two questionnaires. There is no dominant group, as both children with high and low levels of neophobia can exhibit both positive and negative attitudes toward different types of food.

## 4. Discussion

Food neophobia, or aversion to eating new foods, is common among children and can significantly affect their eating habits. In our study, we found that the majority (42.11%) of children showed a high level of neophobia, which is consistent with the results obtained by Laureati et al. in the FNS scale [22]. According to Koziol-Kozakowska et al., an average level of neophobia was found in 76.9% of children; moreover, no statistically significant differences in CFNS scale scores were observed between boys and girls. For this reason, we did not differentiate the results based on gender [23]. Individuals with high levels of neophobia often exhibit strong preferences for routine and familiarity; pressuring the child to eat was found to be positively correlated.

New things may make them anxious or fearful, leading them to avoid change and new situations. Due to food neophobia in adolescence, neophobic behaviours may be carried over into adulthood, as it has been indicated that such behaviours often remain present from age 13 into adulthood. At the same time, the age of 9 years is indicated as critical, as the development of eating behaviours occurs before this age [24]. According to Halland et al., neophobia can affect dietary variety, as children with high levels of neophobia were less likely to eat varied meals. Nearly 40% of children with neophobia did not eat raw vegetables at all [25]. A study by Maratos et al. suggests that food neophobia in children may initially be more strongly associated with visual perception of food, implying that the appearance of food plays a key role in shaping their willingness to try new foods [26,27,28]. A study by Kaar et al. examined the associations between parents’ eating practices and parent–child food neophobia with children’s food preferences. In a sample of 210 parent–child pairs, pressuring the child to eat was found to be positively correlated with the child’s food neophobia, i.e., increased child’s food neophobia, while offering the child new foods was found to be negatively correlated (i.e., decreased child food neophobia). Moreover, the degree of neophobia in children is associated with their preference for and consumption of diverse foods. Specifically, children who exhibit a greater preference for a variety of foods tend to demonstrate lower levels of food neophobia. In other words, if a child likes different foods, he or she is less afraid to try new foods. The Kaar et al. study is consistent with our results, which show that children are afraid to eat things they have never eaten before or know nothing about [29]. In question 7 of the CFNS regarding the fear of trying new foods, 62% of mothers’ responses indicated a high level of their children’s reluctance toward foods they had not encountered before. Factors associated with food neophobia include the influence of parents, who can shape their children’s eating habits through their own food choices and attitudes toward food. Children’s innate taste preferences, such as a natural inclination toward sweet or salty flavours, also play an important role. In addition, sensory aspects of food, such as the appearance, smell, taste, and texture of foods, and previous positive associations can significantly influence children’s openness to trying new foods [4]. Children show distrust of new foods, which is a significant barrier to their consumption. These findings are consistent with our research, which shows specific reactions to new foods [30,31]. In this case, parental competence plays a key role in supporting children with food neophobia, emphasising the need for knowledge and skills to deal with difficult food-related situations [32,33]. Activities that increase familiarity and contact with food can reduce food neophobia in both children and adults [34]. While neophobia can be overcome through early exposure to a variety of nutrient-rich foods, certain impressions and associations may be too strong for acceptance, such as the case of rabbit meat. Although rabbit meat offers valuable nutritional properties, its consumption in the EU is limited (<3%). Children seem to have a negative attitude toward this type of meat, which may be related to its limited presence in the diet and little knowledge of its health benefits [35]. Another product showing negative feelings is edible insects. As in our study, children show a negative attitude toward eating insects. The Nyberg et al. study revealed that children balance between curiosity and fear, reflecting the tension between neophobia and neophilia. Children often experience mixed feelings, such as fascination and protection, which coexist with fear, disgust, and uncertainty about insects as food [36,37]. Consumer acceptance of edible insects has been found to be low, which may be due to food neophobia and fear of insect consumption [38]. In our study, children also showed a negative attitude toward insects, which confirms the need for observations regarding this phenomenon. The use of the CFNS (Children’s Food Neophobia Scale) provided a tool for assessing food neophobia, and the pictorial questionnaire enabled accurate assessment of children’s attitudes toward various foods. Despite its strengths, parental self-reporting may introduce bias. To address this problem, the study incorporated both parent-reported assessments and children’s direct responses, which were analysed independently. This approach helps to mitigate the impact of parental bias and allows for a more comprehensive understanding of children’s food neophobia. Additionally, future studies could benefit from complementing such methods with direct observation of children’s behaviour or the use of additional assessment tools. A study by Siddiqui et al. found a link between food neophobia and attitudes toward eating insects. These analyses highlight the role of neophobia in shaping aversion to and perceptions of insects. Moreover, they suggest that individuals with higher levels of neophobia show greater aversion to new or unfamiliar foods [39]. In contrast, a study by Szlachciuk et al. indicates that individuals with lower levels of food neophobia are more open to experimenting with unconventional foods, such as insects [40]. Similar findings are presented by Yalçın et al., emphasising that more neophobic individuals experience greater fear or aversion to trying new foods [41]. A two-dimensional analysis showed that among those with food neophobia, the percentage of individuals with lower levels of openness is more than four times higher than among those who are more open. In contrast, among those with food neophilia, only one in seven individuals has a lower level of openness, while the rest are characterised by greater openness [42]. Individuals with higher levels of food neophobia are less inclined to purchase new food items. They tend to prefer not only familiar foods but also products that are locally produced [43]. Moreover, according to a study by E. Faccio et al., higher levels of neophobia correlate with lower willingness to eat healthy foods in children [44]. Although the study by Siddiqui et al. [39] demonstrated a relationship between food neophobia and attitudes toward insect consumption, our study did not confirm this association. No correlation was found between the two questionnaires. According to Julyana Nogueira Firme et al., food neophobia does not change with age, suggesting the persistence of this behaviour and potential nutritional challenges in adulthood. Therefore, a special focus on this group is needed to minimise long-term consequences [45]. de Almeida et al. additionally stated that, on the contrary, neophobia can increase with age and intensely affects the consumption of healthy food, and it is a major barrier to consuming fruits and vegetables [46]. Food avoidance or dietary selectivity in childhood can lead to numerous health consequences, both physical and psychological, in adulthood. The treatment of food neophobia in the psychodietetic approach is mainly based on the gradual introduction of new foods, taking into account their taste, texture, and appearance, and the systematic expansion of the diet. An important part of the therapy is also working with parents and carers to help them better understand the phenomenon, which promotes a smoother course of the problem [47]. Pearson’s Chi2 test was conducted in this regard, and the results suggest that there is no statistically significant relationship between the level of neophobia and children’s attitudes towards the food products assessed (*p* > 0.05). This indicates that the CFNS results do not significantly influence the reported attitudes of children towards food. Further research should focus on analysing the environmental and family factors that may mitigate the intensity of neophobia, as well as educational intervention that supports children in overcoming their fear of novel foods. It is important to examine how various approaches for introducing new foods might influence the long-term development of healthy eating habits. For instance, methods such as gradual exposure to unfamiliar foods, offering positive reinforcement when children try new items, and involving them in meal preparation could help mitigate food neophobia. Furthermore, educational programmes aimed at parents and caregivers, focusing on how to foster positive food experiences and promote a diverse diet, could further encourage healthy eating patterns in children. Implementing these strategies may assist children in developing more favourable attitudes toward new foods, thereby supporting the sustained formation of appropriate eating habits. The study’s limitations include potential reporting bias in parental assessments using the CFNS, as parents may overestimate or underestimate their child’s neophobia. Additionally, while the pictorial questionnaire for children facilitated understanding, it may not fully capture the complexity of their attitudes, especially if social desirability influenced responses. The cross-sectional nature of the study also limits the ability to infer causality in the development of food neophobia. Future research should consider longitudinal studies and observational methods to gain a deeper understanding of these factors.

## 5. Conclusions

In the studied population, 42.11% of children exhibited high food neophobia, which raises concerns about their limited dietary variety. This may lead to nutritional imbalances, as these children are less likely to try new foods and may rely on a narrow range of familiar foods that do not support healthy growth. These findings emphasise the need for early interventions to encourage food variety and promote balanced eating habits. These children exhibited a pronounced reluctance to try new foods, which may lead to a limited dietary variety and potential nutrient deficiencies. The smallest group consisted of children with a low level of neophobia (32.33%), who were the most open to new flavours and food products. The analysis of CFNS results revealed significant differences among the three groups, confirming that children with high neophobia were more likely to reject novel foods, whereas those with low neophobia were more inclined to experiment and try different types of food. Among the 12 food products assessed by children, the most accepted were familiar and commonly consumed items, such as ice cream, tomatoes, and cutlets, while edible insects and edible flowers were the most rejected. Statistical analysis did not reveal a significant correlation between the level of neophobia assessed by parents (CFNS) and the actual attitudes of children toward different groups of food products. This indicates that parental self-assessment of their child’s neophobia does not directly translate into the child’s actual food choices. This lack of correlation suggests that the way parents perceive their child’s neophobia may not always reflect the child’s actual willingness to try new foods. Future research should consider incorporating direct behavioural observations or experimental food exposure methods to better assess children’s real attitudes toward novel foods. Given the high proportion of children exhibiting reluctance toward novel foods, there is a need for educational programmes targeted at both children and their parents. These initiatives could help reduce fear of new foods and support the development of a more diverse diet. Since food neophobia did not show a strong correlation with children’s declared attitudes, future research should consider the impact of additional factors, such as the home environment, parental feeding practices, and peer influence, on the formation of children’s eating habits. Future research should focus on exploring how specific factors in the home environment, such as the availability and variety of foods, as well as the frequency of family meals, may influence children’s food preferences and attitudes toward new foods. Parental feeding practices, including pressure to eat, food rewards, or restriction of certain foods, could also play a significant role in shaping children’s willingness to try unfamiliar foods. Additionally, peer influence, especially in social settings like school or group activities, might impact children’s food choices and their perception of novel foods. Understanding the interactions between these factors could provide valuable insights into effective strategies for reducing food neophobia in children.

## Figures and Tables

**Table 1:** Children’s level of neophobia.

	Group [n]	[%] of Population	Group Range [Points]
**Group I (low level of neophobia)**	43	32.33%	<39
**Group II (neutral level of neophobia)**	34	25.56%	38–45
**Group III (high level of neophobia)**	56	42.11%	>46
**All**	133	100%	

CFNS, Child Food Neophobia Scale; *p* < 0.001, Kruskal–Wallis test.

**Table 2:** Child Food Neophobia Scale.

		All n = 133	Group I n = 43	Group II n = 34	Group III n = 56
		x^−^ [SD]	x^−^ [SD]	x^−^ [SD]	x^−^ [SD]
**FNS 1**	My child tries new and different foods all the time	3.07 [1.90]	1.44 [0.50]	2.24 [0.85]	4.82 [1.54]
**FNS 2**	My child does not trust new foods	4.29 [1.22]	4.11 [1.90]	4.06 [0.60]	4.57 [0.66]
**FNS 3**	If my child does not know what is in a food, I will not try it	5.45 [1.38]	4.27 [1.37]	6.76 [0.60]	5.55 [0.89]
**FNS 4**	My child likes foods from different countries	3.25 [1.78]	1.65 [0.78]	2.82 [0.67]	4.75 [1.57]
**FNS 5**	My child thinks ethnic foods are too strange to eat	4.90 [1.32]	3.47 [0.83]	5.56 [1.02]	5.61 [0.82]
**FNS 6**	My child will try new foods	3.32 [2.08]	1.40 [0.49]	2.47 [0.99]	5.32 [1.47]
**FNS 7**	My child is afraid to eat things he/she has never eaten before	5.24 [1.53]	4.16 [1.98]	5.47 [0.75]	5.93 [0.95]
**FNS 8**	My child pays special attention to what he/she will eat	5.00 [1.71]	3.40 [1.89]	5.44 [1.11]	5.96 [0.71]
**FNS 9**	My child will eat almost anything	3.00 [1.79]	1.70 [0.89]	2.24 [0.92]	4.46 [1.64]
**FNS 10**	My child likes to try new ethnic restaurants	4.68 [2.27]	1.83 [1.19]	5.71 [1.06]	6.25 [1.08]
	Mean	42.21	27.47	42.71	53.23

x^−^, mean; SD, standard deviation; group I (low level of neophobia), group II (neutral level of neophobia), group III (high level of neophobia); Child Food Neophobia Scale; *p* < 0.05, Kruskal–Wallis test.

**Table 3:** Level of children’s attitudes towards products.

	Group	[%] of Population	Category Range
Group I (positive attitude)	58	43.61%	37–60
Group II (negative attitude)	52	39.10%	12–33
Group III (ambivalent attitude)	23	17.29%	34–36

**Table 4:** Children’s preferences for suggested foods.

Lp.	Product	I Really Like for n Children (5 Points)	Neither Like nor Dislike (3 Points) n Children	I Do not Like Very Much (1 Point) for n Children	Point Value
1.	Shrimp	20	22	91	1.78
2.	Insect	0	3	130	1.39
3.	Flower	0	10	123	2.56
4.	Rabbit	13	17	103	2.28
5.	Egg	70	21	42	3.29
6.	Cutlet	107	26	0	4.47
7.	Cheese	11	13	109	1.53
8.	Tomato	120	13	0	3.77 *
9	Ice cream	133	0	0	4.20 *
10.	Hamburger	65	25	43	3.86 *
11.	Ketchup	58	31	44	3.29 *
12.	Pasta	59	34	40	3.30 *

K–W test *p* < 0.05; * statistically significant differences between the three groups of children’s attitudes towards 12 food products.

## Data Availability

All data and materials are included in the manuscript.

## Supplementary Materials

The following supporting information can be downloaded at https://www.mdpi.com/article/10.3390/nu17081347/s1, Figure S1: Graphic questionnaire for attitude assessment.
