# Supplementary material for: The Relationship Between the Level of Food Neophobia and Children’s Attitudes Toward Selected Food Products
Source: Nutrients. 2025 Apr 15;17(8):1347. doi: 10.3390/nu17081347 (PMC12029880; doi:10.3390/nu17081347)

Figure S1: Graphic questionnaire for attitude assessment

A questionnaire aimed at children, consisting of 12 pictorial questions on food preferences for various products. Each question is illustrated with relevant pictures to make it easier for children to understand the content, as well as to increase their involvement in completing the survey.
The survey is fully anonymous and the results will be used for research purposes only.
The duration of the questionnaire is about 7 minutes.
The questionnaire is completed by the child.
<u>Rate your feelings about the featured photo by choosing one of three faces:</u>

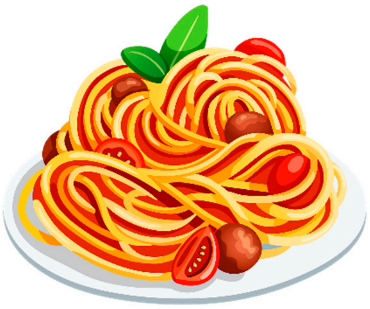

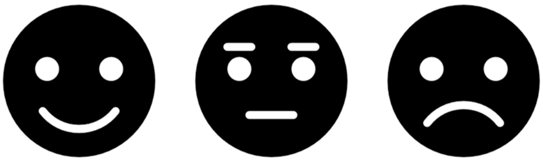

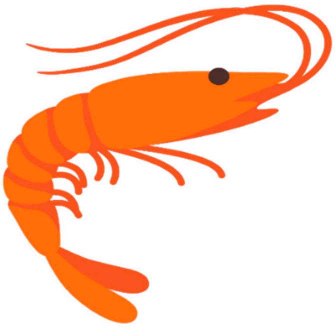

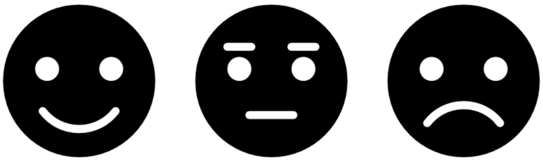

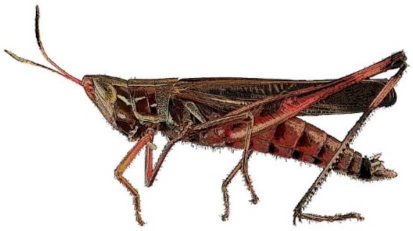

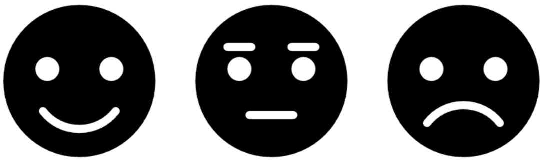

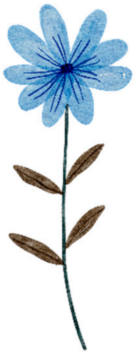

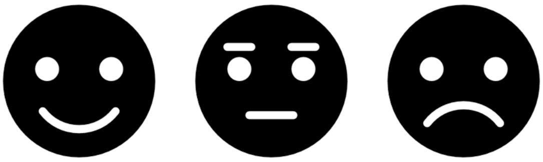

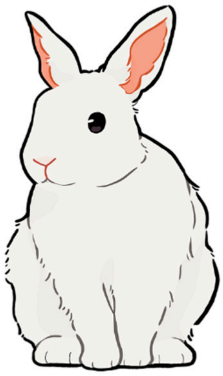

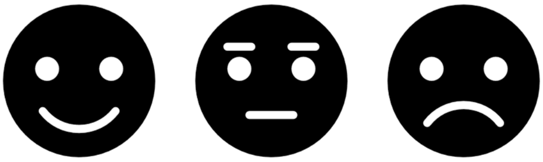

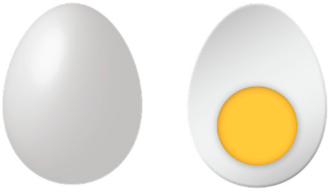

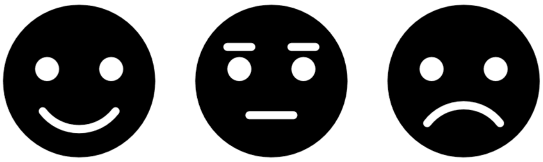

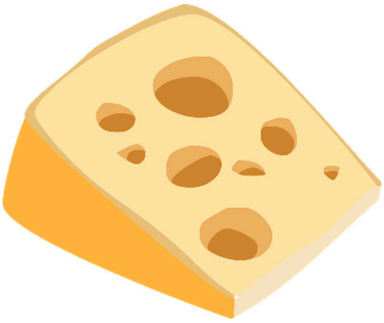

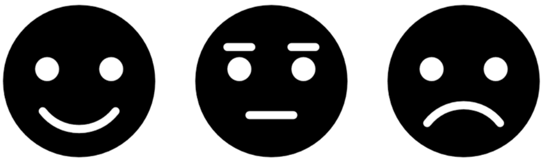

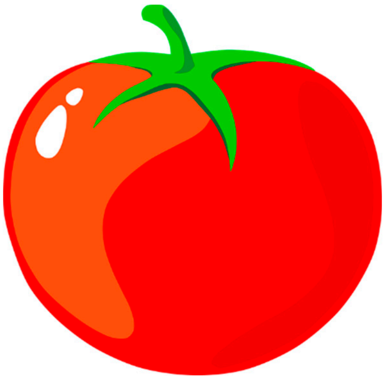

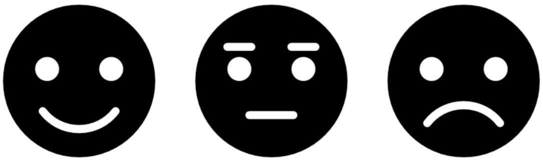

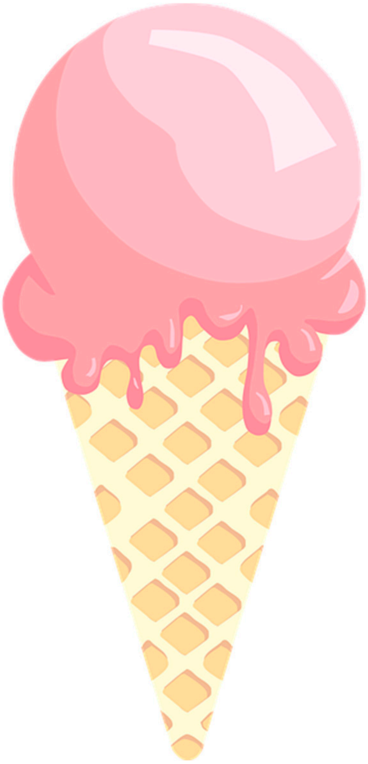

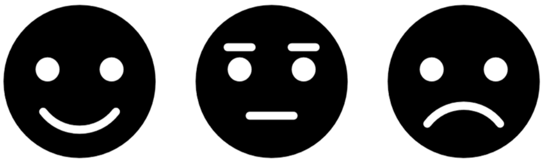

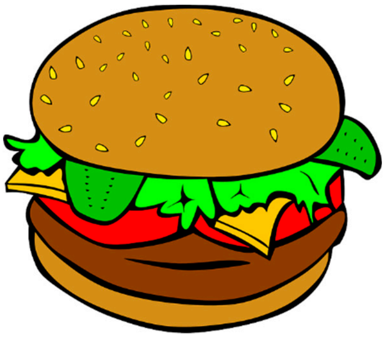

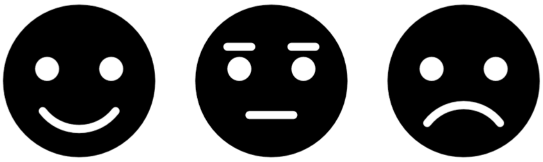

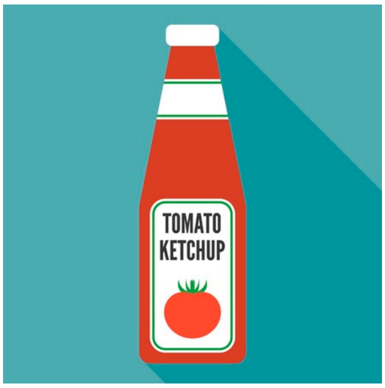
TOMATO
KETCHUP

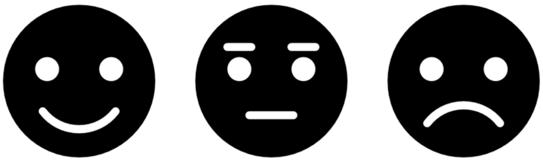

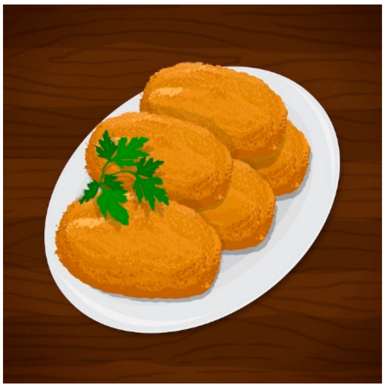

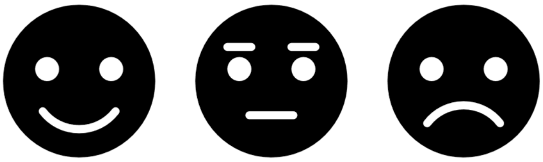

Supplement: Supplementary file 1 [file nutrients-17-01347-s001.zip › nutrients-3568533-supplementary.pdf]
